# Dysregulation of Innate Lymphoid Cells in Patients with Active Rheumatoid Arthritis and Mice with Collagen-Induced Arthritis

**DOI:** 10.1155/2021/1915068

**Published:** 2021-02-20

**Authors:** Fengfan Yang, Xing Luo, Wenxiao Zhu, Jia Li, Zhaohui Zheng, Ping Zhu

**Affiliations:** ^1^Department of Clinical Immunology, PLA Specialized Research Institute of Rheumatology & Immunology, Xijing Hospital, Fourth Military Medical University, No. 127 Changle West Road, Xi'an, 710032 Shaanxi, China; ^2^National Translational Science Center for Molecular Medicine, Fourth Military Medical University, No. 169 Changle West Road, Xi'an, 710032 Shaanxi, China

## Abstract

Innate lymphoid cells (ILCs) have roles in many diseases and immune pathways. To determine the roles of these cells in patients with rheumatoid arthritis (RA) and mice with collagen-induced arthritis (CIA), we measured ILC subsets using flow cytometry and multiplex immunofluorescence staining. Patients with stable RA had greater proportions of ILC2s and decreased proportions of ILC1s and ILC3s (all *p* < 0.05). The 28-joint disease activity (DAS28) score had positive correlations with the proportion of ILC1s and negative correlations with ILC2s (both *p* < 0.05). ILC2s of patients with RA expressed more IL-4 than healthy controls (*p* < 0.05). The proportions of ILC1s and ILC2s were greater in mice with CIA (both *p* < 0.05), especially the ILC2s in mice without arthritis after immunization and had correlations with multiple inflammatory and anti-inflammatory cytokines. Multiplex immunofluorescence staining described the distribution of ILCs in spleen tissues. Our results indicate that dysregulation of ILCs occurs during the pathogenesis of RA and CIA.

## 1. Introduction

Innate lymphoid cells (ILCs) are immune cells that do not express rearranged antigen-specific receptors, but nonetheless exhibit a variety of important immunological functions. ILCs can be divided into three categories based on the presence of transcription factors and cytokines: ILC1s include conventional natural killer (cNK) cells, express T-bet, and secrete IFN-*γ*; ILC2s express GATA3, Bcl11b, and GF1 and secrete IL-4, IL-5, IL-9, and IL-13 and amphiregulin (AREG); and ILC3s express ROR*γ*t and secrete IL-17A, IL-17F, and IL-22. These three categories of ILCs are presumably the noncytotoxic counterparts of CD4+ T cells [1]. During infections, inflammatory responses, and autoimmune diseases, ILCs and the adaptive immune system have interrelated functions and interact with each other [2].

Several studies have indicated that ILCs have a role in autoimmune diseases. For example, one study reported that patients with systemic sclerosis (SSc) had an increased abundance of ILC2s in the derma and in circulation and that ILC2 count correlated with disease score and pulmonary fibrosis [3]. Another study found that the levels of CD4+ILC1 and NKp44+ILC3 cells were increased in the peripheral blood of patients with SSc and that IL-6R*α* expression by CD4+ILC1 cells was related to the therapeutic outcome of tocilizumab treatment [4].

Rheumatoid arthritis (RA) is a chronic inflammatory autoimmune disease characterized by painful and swollen joints, cartilage, and bone damage and eventually leads to severe disability. Researchers now consider Th cells, B cells, monocyte macrophages, cytokines, and autoantibodies to function in the pathogenesis of RA [5–8]. ILCs also appear to function in the pathogenesis of RA, but there are some inconsistent results on this topic. There is evidence that ILCs were present in the synovial fluid (SF) of patients with RA and increased lymphocyte infiltration into the SF of these patients correlated with the amount of lymphoid tissue-inducer- (LTi-) like cells [9]. Another study showed no differences in the amounts of total ILCs in RA patients and healthy controls, but that RA patients had reduced levels of lymphoid tissue-induced (LTi) cells and increased levels of ILC1s and ILC3s. These studies suggest that ILCs might function in the pathogenesis of RA [10].

In this study, we first determined the levels of ILC subsets in the circulation of patients with RA and the relationship of different ILCs with the extent of inflammation and clinical disease index. We then investigated alterations of ILC subsets in mice with collagen-induced arthritis (CIA).

## 2. Materials and Methods

### 2.1. Patients and Controls

Fifty-six patients with RA who met the 2010 ACR/EULAR classification criteria for RA were recruited from the Department of Clinical Immunology of Xijing Hospital. Patients were classified as having active disease status (Disease Activity Score − 28 (DAS28) > 3.2, *n* = 25) or low disease status (DAS28 ≤ 3.2, *n* = 31). All laboratory data were from the clinical laboratory reports. Samples from age- and sex-matched healthy controls (HCs, *n* = 36) were from volunteers who had no abnormalities based on routine physical examinations, no symptoms of arthritis, and no family history of any rheumatic disease. This study was approved by the Ethics Committee of Xijing Hospital and was conducted in compliance with the Declaration of Helsinki. All subjects signed informed consent agreements.

### 2.2. Mice

Female C57BL/6J mice (8 to 10 weeks old) were purchased from Charles River Laboratory (Beijing, China) and were bred and maintained under specific pathogen-free (SPF) conditions. All experiments followed the principles of *Guide for the Care and Use of Laboratory Animals* (NIH) and were approved by the Experimental Animal Care Committee of the Forth Military Medical University (Xi'an, China).

### 2.3. Induction of Collagen-Induced Arthritis

Chicken type II collagen (CII, Chondrex) was dissolved at 4 mg/mL in 0.1 M acetic acid overnight at 4°C and then mixed with an equal volume of 5 mg/mL complete Freund's adjuvant (CFA, Chondrex) using an ultrasonic homogenizer on ice. An emulsion (100 *μ*L) was injected intradermally at the base of the tail of each mouse, and a booster injection with an emulsion of CII in incomplete Freund's adjuvant (IFA, Chondrex) was given 14 days later [11]. Disease scores of mice with CIA were measured by the extent of paw redness and swelling, as described previously [11]. Grade 0 indicated no abnormality, grade 1 indicated slight swelling or redness, grade 2 indicated obvious swelling, and grade 3 indicated severe swelling and ankyloses. For each mouse, the total score of all four limbs was its total clinical score (maximum of 12) [11].

### 2.4. Flow Cytometry

For samples from RA patients and HCs, anticoagulant peripheral blood was added to red cell lysis buffer (BD Biosciences) and then stained with antibodies from BD Biosciences or BioLegend for 20 min at 4°C: anti-human CD3 (OKT3), CD16 (3G8), CD56 (HCD56), CD4 (RPA-T4), CD8 (RPA-T8), CD19 (HIB19), CD20 (2H7), CD14 (M5E2), CD34 (581), CD123 (6H6), CD94 (DX22), HLA-DR (L243), Fc*ε*R1*α* (AER-37, all FITC conjugated), CD3-BV421 (OKT3), CD16-BV711 (3G8), CD56-APC/cy7 (HCD56), CD45-BV570/BV650 (HI30), CD161-PE/Dazzle594 (HP-3G10), CD127-BV605 (A019D5), CD294-PE/cy7 (BM16), and CD117-PE/cy5 (A3C6E2). The lineage markers were CD3, CD4, CD8, CD19, CD20, CD14, CD16, CD56, CD34, CD123, CD94, HLA-DR, and Fc*ε*R1*α*. For intracellular staining, blood samples were incubated with cell activation cocktail (Biolegend) at 37°C in a CO_2_ incubator for 4 h. After surface staining, cells were fixed and permeabilized using the Cytofix/Cytoperm Kit (BD Biosciences), and intracellular cytokines were then stained with different antibodies for 30 min at room temperature: IFN-*γ*-BV711 (4SB3), IL-13-BV711 (JES10-5A2), IL-9-PE (MH9A3), IL-4-APC (8D4-8), and IL-17A-BV421 (BL168). Precision count beads (BioLegend) were used to determine absolute cell counts.

For staining of mouse cells, the spleen and inguinal lymph nodes were removed, milled, and passed through a 70 *μ*m strainer. After centrifugation (300 g, 5 min), cells were resuspended in a phosphate buffer (pH 7.2). Red blood cells from the spleen were suspended in red cell lysis buffer. For surface staining, splenocytes and lymphoid node cells were incubated in a solution of antibodies from BD Biosciences or BioLegend for 20 min at 4°C: anti-mouse CD45-BV570 (30-F11), Lineage-PerCP-cy5.5 (17A2/RB6-8C5/RA3-6B2/Ter-119/M1/70), and CD127-BV421 (A7R34). Cells were treated with Foxp3/Transcription Factor Staining Buffer Set (eBioscience), and then, Eomes-AF647 (W17001A), T-bet-PE-cy7 (4B10), GATA3-PE (16E1A23), and ROR*γ*t-PE (B2D) were added for transcription factor staining.

The processed cells were analyzed using a SONY SP6800 Spectral Analyzer or a Beckman Coulter-CytoFLEX flow cytometer and corresponding software.

### 2.5. Detection of Cytokines in Mouse Serum

Blood was collected from the tails of mice each week without anticoagulants, centrifuged for 5 min at 8000 rpm, and the serum was then removed and stored at –30°C. Cytokine levels were measured using the LEGENDplex Mouse Inflammation Panel and the Th Cytokine Panel (BioLegend) according to the manufacturer's instructions. Samples were first diluted 2-fold. Then, 25 *μ*L of assay buffer and 25 *μ*L of the diluted sample or standards with 25 *μ*L of mixed beads were added to each well of a culture plate. The plate was protected from light and agitated (800 rpm for 2 h) at room temperature and then rinsed twice with a washing buffer. Then, 25 *μ*L of the antibody solution was added to each well and the plate was agitated (800 rpm for 1 h). Then, 25 *μ*L of streptavidin phycoerythrin (SA-PE) was added to each well, the plate was agitated (800 rpm for 30 min) and washed, and the beads were resuspended. The samples were then detected using a Beckman Coulter-CytoFLEX flow cytometer. FCS files were analyzed using LEGENDplex Data Analysis software.

### 2.6. Multiplex Immunofluorescence

Paraffin sections of the mouse spleen were deparaffinized and rehydrated following routine procedures. For staining with different antigens, slides were incubated with an appropriate primary antibody, followed by a horseradish peroxidase- (HRP-) conjugated secondary antibody. The fluorochrome was then added, and the fluorescent dye directly combined with the antigen epitope (tyramine signal amplification technique). Microwave heating was used to remove the nonspecific binding secondary antibody and flourochrome, and samples were than processed for the next staining cycle. Multiplex immunofluorescence was achieved using several rounds of sequential antibody staining. Nuclei were stained with 4′-6′-diamidino-2-phenylindole (DAPI) on the final step. The primary antibodies were from Abcam or Cell Signaling Technology: anti-CD127 (EPR2955 [2]), CD3e (D4V8L), CD19 (D4V4B), F4/80 (D2S9R), T-bet (4B10), GATA3 (1A12-1D9), and ROR*γ*t (EPR20006). The lineage markers include CD3e, CD19, and F4/80. The PANO 7-plex IHC kit, secondary antibodies, fluorescent dyes, and DAPI were purchased from Panovue.

The stained slides were scanned using the PerkinElmer Mantra system, and inForm image analysis software was used for measurements of the different dyes and autofluorescence. The extracted images were analyzed using HALO software.

### 2.7. Statistical Analysis

Data were presented as means ± SDs, and comparisons between two groups were performed using Student's *t*-test or Fisher's exact test, as appropriate. Correlations were calculated using Spearman's rank correlation analysis. All statistical analyses were performed using GraphPad Prism version 5. A *p* value below 0.05 was considered significant.

## 3. Results

### 3.1. RA Patients with Active Disease Have More Alterations of Disease Markers than Those with Stable Disease

We classified the 56 RA patients as having active disease status (DAS28 > 3.2) or low disease status (DAS28 ≤ 3.2, Table 1). These two groups had no significant differences in mean age, sex ratio, disease duration, rheumatoid factor (RF) positivity, and anticyclic-citrullinated peptide (CCP) positivity (all *p* > 0.05). However, patients with active disease status had significantly greater levels of CRP and ESR, more tender and swollen joints, greater visual analogue scale (VAS) score for pain, and greater DAS28 score (all *p* < 0.001).

### 3.2. RA Patients Have Altered Proportions of ILC1s and ILC2s and This Alteration Correlates with DAS28

**Figure 1:**
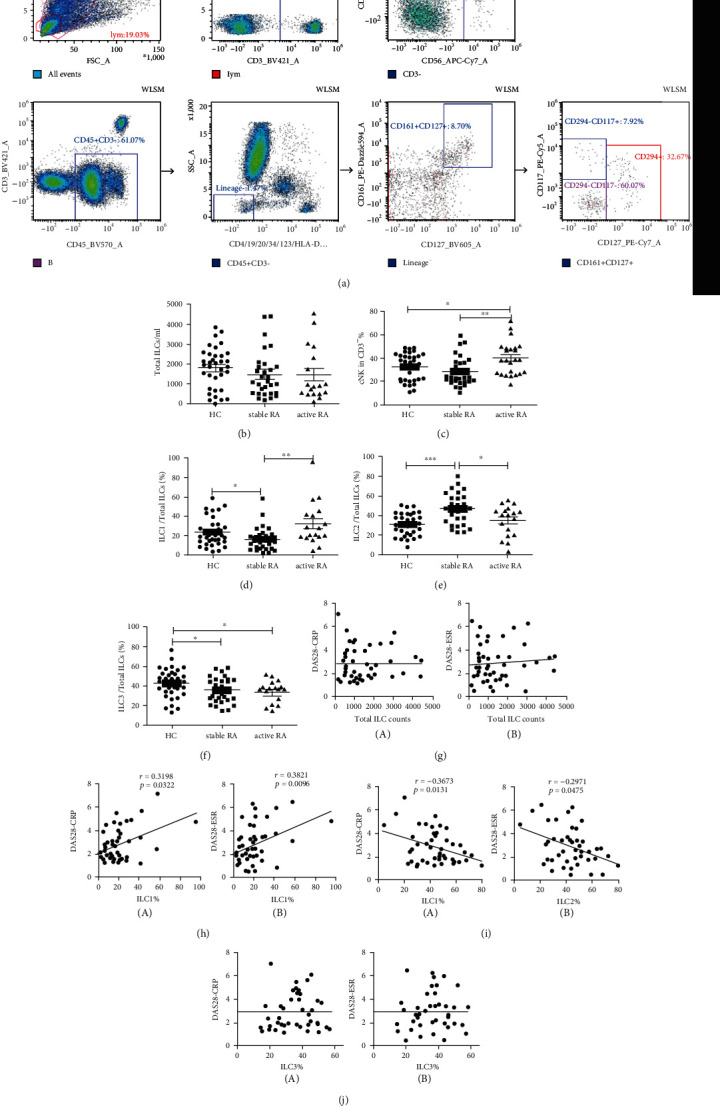
ILC subsets in the peripheral blood of HCs, patients with stable RA, and patients with active RA. (a) Gating strategy for total ILCs (CD45+CD3-Lineage-CD161+CD127+), cNK cells (CD3-CD16+/CD56+), ILC1s (CD294-CD117-), ILC2s (CD294+CD117-), and ILC3s (CD294-CD117+). (b) Absolute counts of total ILCs per mL of peripheral blood. (c) Percentage of CD16+/CD56+ cNK cells among CD3- cells. (d–f) Proportions of ILC1s, ILC2s, and ILC3s among total ILCs. (g) Correlations of the total number of ILCs with DAS28-CRP (A) and with DAS28-ESR (B). (h–j) Correlations of proportions of ILC1s, ILC2s, and ILC3s in total ILCs with DAS28-CRP (A) and DAS28-ESR (B). Data are expressed as means ± SDs and compared using the unpaired Student's *t*-test. Spearman's rank correlation coefficients and *p* values are indicated. ^∗^*p* < 0.05, ^∗∗^*p* < 0.01, and ^∗∗∗^*p* < 0.001. RA: rheumatoid arthritis; HC: healthy control; DAS28: disease activity score for 28 joints; CRP: C-reactive protein; ESR: erythrocyte sedimentation rate. HC: *n* = 36; stable RA: *n* = 31; and active RA: *n* = 25.

We measured ILCs in the peripheral blood of 56 RA patients and 39 HCs (Figure 1(a)). The results indicated that the RA patients and HCs had similar absolute numbers of total ILCs (CD45+CD3-Lineage-CD161+CD127+) (Figure 1(b)), but the percentages of cNK cells (CD3-CD16+/CD56+) were significantly greater in RA patients with active disease status than in those with stable disease status and HCs (Figure 1(c)). In addition, we examined ILC1s (CD294-CD117-), ILC2s (CD294+CD117-), and ILC3s (CD294-CD117+). Patients with stable RA had a decreased proportion of ILC1s and an increased proportion of ILC2s relative to those with active RA and HCs (Figures 1(d) and 1(e)). RA patients had a decreased proportion of ILC3s relative to HCs (Figure 1(f)). The correlations of the absolute number of total ILCs and the proportion of ILC3s with disease activity were not significant (Figures 1(g) and 1(j)). However, the proportion of ILC1s had a positive correlation with disease activity (Figure 1(h)). There was a negative correlation between the proportion of ILC2s and disease activity (Figure 1(i)).

**Figure 2:**
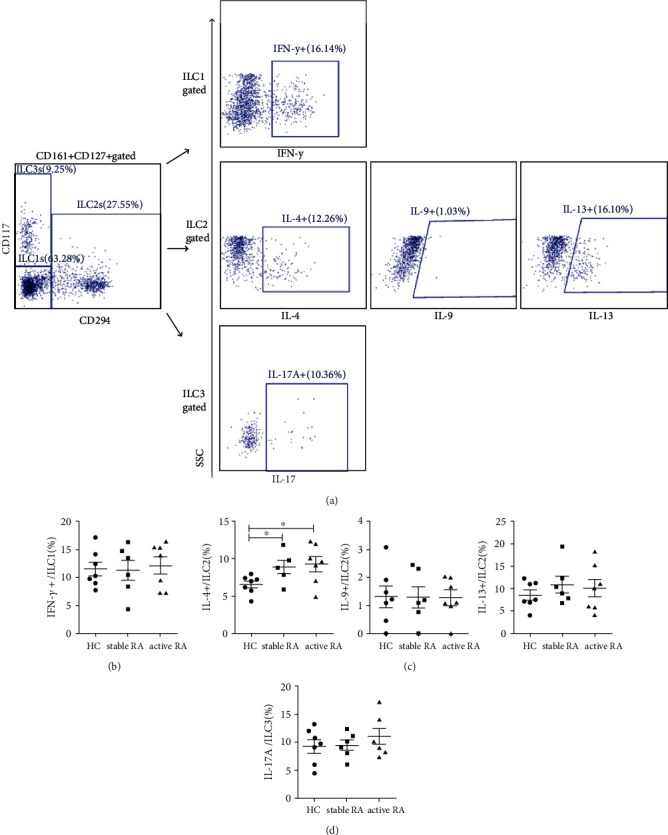
Cytokine expression by ILC subsets in the peripheral blood of HCs and patients with RA. (a) Gating strategy for IFN-*γ*, IL-4, IL-9, IL-13, and IL-17A in ILC subsets. (b) Percentage of IFN-*γ*+ cells in ILC1s. (c) Percentage of IL-4+, IL-9+, and IL-13+ cells in ILC2s. (d) Percentage of IL-17A+ cells in ILC3s. Data are expressed as means ± SDs and compared using the unpaired Student's *t*-test. ^∗^*p* < 0.05. RA: rheumatoid arthritis; HC: healthy control; IFN-*γ*: interferon-*γ*; IL: interleukin. HC: *n* = 7; stable RA: *n* = 6; and active RA: *n* = 7.

We also measured the expression of IFN-*γ*, IL-4, IL-9, IL-13, and IL-17A in ILC subsets (Figure 2(a)). The expression of IL-4 by ILC2s was greater in patients with RA than in HCs (Figure 2(c)), but the expressions of IFN-*γ*, IL-9, IL-13, and IL-17A by ILC1s, ILC2s, and ILC3s were not altered significantly (Figures 2(b)–2(d)).

### 3.3. Proportions of ILC1s and ILC2s Are Greater in Mice with CIA

**Figure 3:**
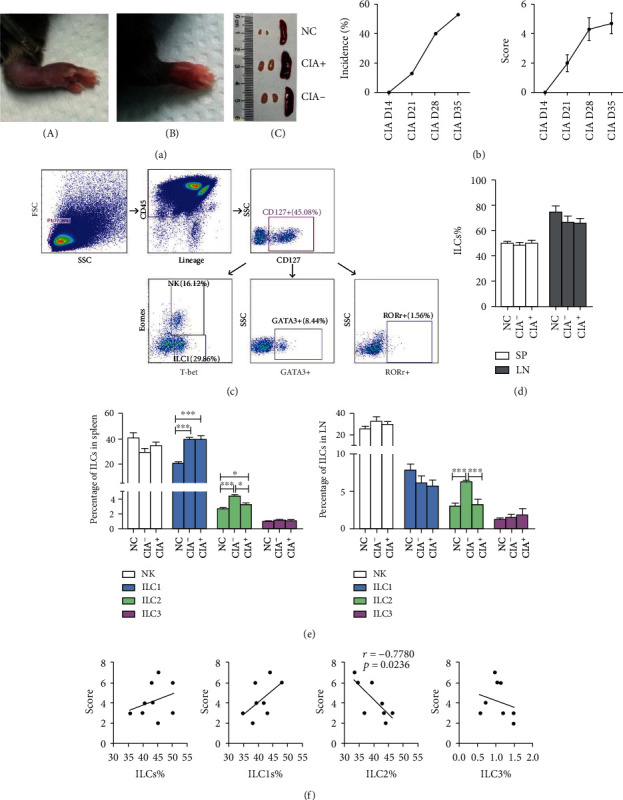
ILC subsets in mice with CIA and NCs. (a) Redness and swelling of paws after immunization (A, B). Lymph nodes and spleens that were obviously enlarged (C). NC: negative control; CIA−: immunized mice without arthritis; CIA+: immunized mice with arthritis. (b) The incidence and disease scores of CIA mice. A total of 17 mice received immunization, and 9 developed arthritis at week-5. (c) Gating strategy for total ILCs (CD45+ Lineage-CD127+), NK cells (T-bet+Eomes+), ILC1s (T-bet+Eomes-), ILC2s (GATA3+) and ILC3s (ROR*γ*+). (d, e) Proportion of total ILCs, NK cells, ILC1s, ILC2s, and ILC3s in the spleens and lymphoid nodes of mice with CIA and NCs. NC, *n* = 5; CIA−, *n* = 5; and CIA+, *n* = 9. (f) Correlation of the proportion of ILC subsets in the spleen with disease score at week-5. NC: negative control; CIA: collagen-induced arthritis; SP: spleen; LN: lymph nodes. Data are expressed as means ± SDs and analyzed using the unpaired Student's *t*-test. Spearman's rank correlation coefficients and *p* values are indicated. ^∗^*p* < 0.05, ^∗∗∗^*p* < 0.001.

The mice began to develop arthritis three weeks after the initial immunization, and we recorded the incidence and disease scores based on paw redness and swelling (Figures 3(a) and 3(b)). Immunized mice were divided into two groups (CIA−: without arthritis; CIA+: with arthritis) based on whether they had arthritis during immunization. Five weeks after the first injection with type CII, we euthanized mice for collection of the spleens and lymphoid nodes to evaluate the ILC subsets (Figure 3(c)). We identified total ILCs as CD45+ Lineage-CD127+ and further classified the cells as NK cells (T-bet+Eomes+), ILC1s (T-bet+Eomes-), ILC2s (GATA3+), or ILC3s (ROR*γ*t+) [12]. The CIA mice and negative control (NC) mice had similar proportions of total ILCs, NK cells, and ILC3s (Figures 3(d) and 3(e)). However, the CIA− and CIA+ mice had higher proportions of ILC1s in their spleens than the NC mice; CIA− mice had a higher proportion of ILC2s in their spleens and lymphoid nodes than NC and CIA+ mice; and CIA+ mice had a higher proportion of ILC2s in their spleens than NC mice (Figure 3(e)).

The correlations between the proportion of total ILCs, ILC1s, and ILC3s with CIA disease score were not significant in the CIA+ mice, but the proportion of ILC2s had negative relationship with the disease score (Figure 3(f)).

**Figure 4:**
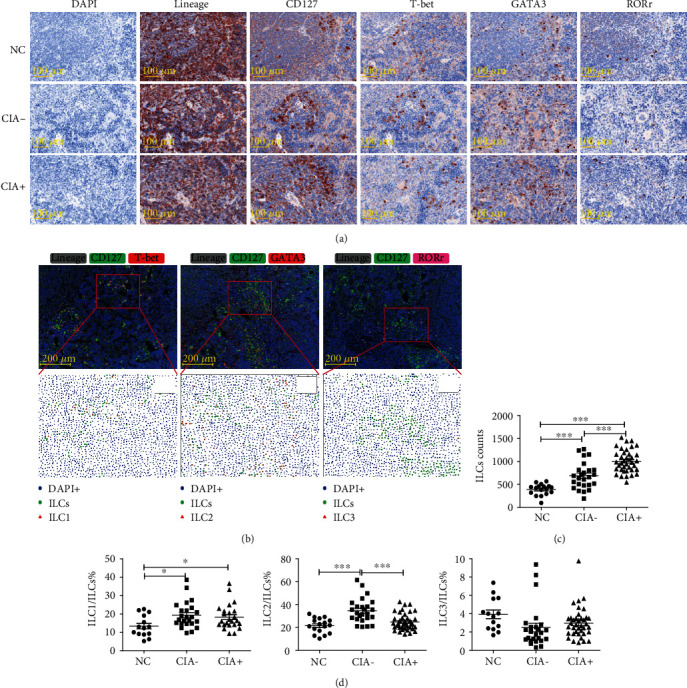
ILC subsets in the spleens of mice with CIA and NCs. (a) Extracted single spectrum of each labeled marker from immunofluorescence staining shown as an immunohistochemistry image. (b) Multiple fluorescence image (top) and phenotyping analysis by HALO software (bottom), in which each symbol represents a phenotype (blue dots: total cells (DAPI+); green dots: total ILCs (Lineage-CD127+); red triangle (left): ILC1s (Lineage-CD127+T-bet+), red triangle (middle): ILC2s (Lineage-CD127+GATA3+); red triangle (right): ILC3s (Lineage-CD127+ROR*γ*t+)). (c) Numbers of total ILCs in each field of view. (d) Proportions of ILC1s, ILC2s, and ILC3s among total ILCs. Each point represents a random observation from a spleen section from 2 to approximately 4 mice per group. NC: *n* = 17; CIA−: *n* = 25; and CIA+: *n* = 38. NC: negative control; CIA: collagen-induced arthritis. Data are expressed as means ± SDs and analyzed using the unpaired Student's *t*-test. Spearman's rank correlation coefficients and *p* values are indicated. ^∗^*p* < 0.05, ^∗∗∗^*p* < 0.001.

We also assessed the distribution of ILCs in spleen tissues (Figures 4(a) and 4(b)). Immunofluorescence staining showed that the total numbers of ILCs (Lin-CD127+) in each field of view (0.64 mm^2^) were obviously increased in all CIA mice, especially the CIA+ mice (Figure 4(c)). The alteration of ILC1s was similar to the results from flow cytometry; the CIA− mice had greater proportions of ILC2s than the NC and CIA+ mice (Figure 4(d)). The proportion of ILC3s did not change.

### 3.4. Proportion of ILC2s Correlates with the Levels of Multiple Cytokines in Mice with CIA

**Figure 5:**
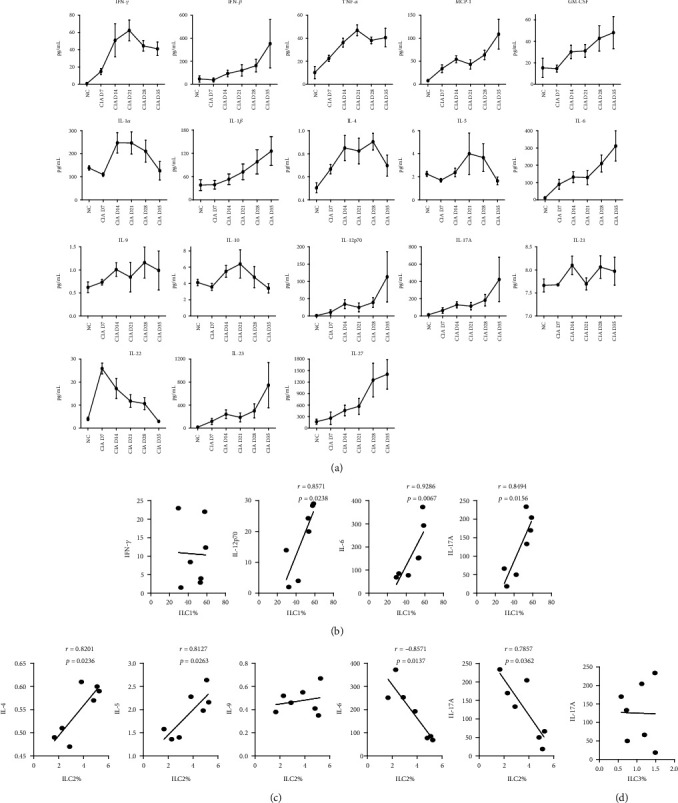
Relationship of ILCs and inflammation-associated cytokines in mice with CIA. (a) Serum levels of 18 cytokines (IFN-*γ*, IFN-*β*, TNF-*α*, MCP-1, GM-CSF, IL-1*α*, IL-1*β*, IL-4, IL-5, IL-6, IL-9, IL-10, IL-12p70, IL-17A, IL-21, IL-22, IL-23, and IL-27) on day-0, week-1, week-2, week-3, week-4, and week-5. Each time point is the cytokine level in 7 to 10 mice. (b–d) Correlations of cytokines with the proportions ILC1, ILC2s, and ILC3s in the spleens of CIA mice. (b) Correlations of the proportion of ILC1s with IFN-*γ*, IL-12p70, IL-6, and IL-17A. (c) Correlations of the proportion of ILC2s with IL-4, IL-5, IL-9, IL-6, and IL-17A. (d) Correlations of the proportion of ILC3s in peripheral blood with IL-17A. Spearman's rank correlation coefficients and *p* values are indicated. IFN-*γ*: interferon-*γ*; TNF-*α*: tumor necrosis factor-*α*; MCP-1: monocyte chemoattractant protein-1; GM-CSF: granulocyte-macrophage colony-stimulating factor; IL: interleukin. *N* = 7.

We measured the time course of changes in the levels of multiple cytokines in mice with CIA starting on day-0. The results showed that most of the measured cytokines increased after immunization (Figure 5(a)). Correlation analysis of the proportion of ILC subsets in the spleen at week-5 with the levels of different cytokines at the same time showed there were significant and positive correlations of ILC1s with IL-12p70, IL-6, and IL-17A (Figure 5(b)). The ILC2s had positive correlations with IL-4 and IL-5 but had negative correlations with IL-6 and IL-17A (Figure 5(c)). ILC1s and ILC3s had no significant correlation with IFN-*γ* or IL-17A (Figures 5(b) and 5(d)). All of these cytokines have well-established roles in the innate or adaptive immune pathways.

## 4. Discussion

Recent studies of the many functions of ILCs in inflammation and immunity have changed our understanding of immune regulation and homeostasis [2]. It is now well established that different ILC subsets function in the pathogenesis of many diseases. For instance, ILC1s can protect against infection from an intracellular parasite by producing IFN-*γ* and TNF [13], but ILC1s also accumulate in the inflamed intestines of people with Crohn's disease and produce the proinflammatory cytokine IFN-*γ* [14]. ILC2s can produce Th2 cytokines, which promote airway hyperresponsiveness and allergic asthma [15], but ILC2s also upregulate IL-33 expression in mice and humans, and depletion of these cells leads to failure of wound healing, suggesting they have a protective effect in this process [16]. ILC3s can express MHC-II to present microbiota-related antigens to commensal-specific CD4+T cells, leading to cell death and immune tolerance, and this process is dysregulated in patients with inflammatory bowel disease [17], but CCR6+ILC3 cells also accumulate in the inflamed joints of patients with RA and mice with CIA, where they produce IL-17 and IL-22 [18].

In this study, we measured the different ILC subsets in HCs and patients with RA. The absolute number of ILCs was not altered in RA patients relative to HCs, although the proportion of cNK cells was greater in RA patients. Patients with stable RA had a decreased level of ILC1s and an increased level of ILC2s relative to those with active RA and HCs. The proportion of ILC1s had a positive correlation with disease activity, but the proportion of ILC2s had a negative correlation with disease activity. These results suggest that ILC2s might have a protective function in the pathogenesis of RA. A previous study reported an increased level of Lin-ICOS+IL-9+ILC2s in patients with RA who were in clinical remission and that the levels of ILC2s were particularly low in those who had persistent inflammatory disease activity [19]. In our study, there was no increased IL-9 production by ILC2s of patients with RA, possibly because we used a different gating strategy for ILC2s: Lin-CD161+CD127+CD294+CD117-. However, the ILC2s of RA patients expressed more IL-4 than HCs, another known anti-inflammatory cytokine. This indicates that the ILCs might control the progress of RA primarily through adjusting the proportions of ILC subsets and the expression of associated cytokines.

To verify our findings in patients with RA, we also measured the different ILC subsets in the spleen and lymphoid nodes of mice with CIA. Relative to NC and mice that developed arthritis, mice without arthritis after immunization had higher proportions of ILC2s. However, the proportions of ILC1s were increased in all CIA mice. This suggests that although not all mice developed the arthritic phenotype following CII injection, all mice that received these injections experienced changes of ILCs. In other words, mice without obvious symptoms are nonetheless in an inflammatory state, and the increased proportion of ILC2s might help relieve their symptoms. RA patients and mice with CIA had different alterations of ILCs; this might be because RA patients often have complex disease status, with different disease courses, therapies, and medical histories, and CIA cannot fully recapitulate these conditions. Further studies are needed to examine the ILC subsets of CIA mice in remission.

The proportions of total ILCs were not different among groups based on flow cytometry, but immunofluorescence staining demonstrated that the numbers of Lin-CD127+ cells increased significantly in some individual cases. In fact, the ILCs were not evenly distributed in tissues but were mainly located in the white pulp between lymphoid follicles; there was considerable discrepancy of cell densities among these regions, and detection of mixed cell suspensions might make the alteration indiscoverable, accounting for the inconsistency between different methods. The application of multiplex immunofluorescence staining and multispectral imaging would provide a better understanding of the distribution of ILCs in various tissues and valuable information for further study in affected synovial tissues, subcutaneous nodules, and other available specimens.

Our measurements of the changes of serum cytokines over time showed that most of the cytokines had maxima at week-4 to week-5 after immunization. Comparisons of the proportions of ILC subsets and cytokines indicated that the proportion of ILC1s was closely associated with inflammation and responses of the immune system. In other words, a strong autoimmune response is associated with increased production of ILC1s. IL-4 and IL-5, which are effector cytokines of ILC2s, had positive correlations with the proportion of ILC2s, suggesting that the immune regulatory effect of these cells is mediated via IL-4 and IL-5 in mice with CIA. The negative correlation between the proportion of ILC2s with IL-6 and IL-17A further confirmed the negative regulatory role of ILC2s in inflammation and autoimmunity. However, the amount of IFN-*γ* and IL-17A (produced by ILC1s and ILC3s, respectively) was not different between HCs and patients with RA. The serum levels of these cytokines also had no correlation with ILC1s and ILC3s in CIA mice. These results indicate that these two cells might function in local tissue, rather than in circulation.

## 5. Conclusions

In conclusion, our study demonstrated that patients with RA and mice with CIA have altered ILC subsets. The proportion of ILC2s was increased in the peripheral blood of patients with stable RA and in immunized mice without arthritis. These results suggest that the ILC2 subset might play a protective role in the pathogenesis of RA and CIA. The abnormal proportions of ILC1s also suggest that ILC subsets might function in the progression of RA and CIA. Further studies are needed to more precisely elucidate the roles of the different ILC subsets in the pathogenesis of RA.

## Figures and Tables

**Table 1:** Characteristics of HCs, patients with stable RA, and patients with active RA.

Characteristics	HCs (*n* = 36)	RA patients (*n* = 56)	
		Low diseases status (DAS28 ≤ 3.2, *n* = 31)	Active disease status (DAS28 > 3.2, *n* = 25)
Mean age (years)	47.53 ± 3.368	44.43 ± 2.260	50.67 ± 2.968
Sex ratio (male/female)	8/28	6/25	5/20
Diseases duration (years)	NA	7.10 ± 1.27	5.82 ± 1.00
RF positivity (*n* (%))	NA	26 (83.88%)	20 (80.00%)
Anti-CCP positivity (*n* (%))	NA	23 (74.19%)	17 (68.00%)
ESR (mm/h)	NA	6.12 ± 1.28	44.45 ± 12.80
CRP (mg/dL)	NA	11.23 ± 1.47	40.84 ± 8.33
Tender joints	NA	1.27 ± 0.50	13.29 ± 1.91
Swollen joints	NA	0.23 ± 0.09	11.06 ± 1.80
VAS score (0-100 mm)	NA	27.33 ± 3.52	56.84 ± 4.53
DAS 28 score (CRP)	NA	2.01 ± 0.11	4.51 ± 0.24
DAS 28 score (ESR)	NA	1.97 ± 0.16	4.74 ± 0.27
Treatment	NA	28 (90.32%)	19 (76.00%)
None (*n* (%))	NA	3 (9.68%)	6 (24.00%)
Steroid (*n* (%))	NA	9 (29.03%)	4 (16.00%)
NSAIDS (*n* (%))	NA	5 (16.13%)	5 (20.00%)
DMARDS (*n* (%))	NA	27 (87.10%)	13 (52.00%)
TNF-*α* antagonist (*n* (%))	NA	9 (29.03%)	2 (8.00%)

Values (mean ± SD or *n* (%)) were compared using the unpaired Student's *t*-test or Fisher's exact test. HCs: healthy controls; RA: rheumatoid arthritis; DAS28: disease activity score for 28 joints; RF: rheumatoid factor; anti-CCP: anticyclic-citrullinated peptide antibodies; ESR: erythrocyte sedimentation rate; CRP: C-reactive protein; VAS: visual analogue scale score for pain; NSAIDS: nonsteroidal anti-inflammatory drugs; DMARDs: disease-modifying antirheumatic drugs; TNF-*α*: tumor necrosis factor-*α*; NA: not available.

## Data Availability

Data will be made available on request.
